# Psychopathology Factors That Affect the Relationship Between Body Size and Body Dissatisfaction and the Relationship Between Body Dissatisfaction and Eating Pathology

**DOI:** 10.3389/fpsyg.2018.02768

**Published:** 2019-01-11

**Authors:** Juliet K. Rosewall, David H. Gleaves, Janet D. Latner

**Affiliations:** ^1^Department of Psychology, University of Canterbury, Christchurch, New Zealand; ^2^Child and Adolescent Eating Disorders Service, South London and Maudsley NHS Foundation Trust, London, United Kingdom; ^3^School of Psychology, Social Work and Social Policy, University of South Australia, Adelaide, SA, Australia; ^4^Department of Psychology, University of Hawaii, Honolulu, HI, United States

**Keywords:** body dissatisfaction, eating pathology, BMI, psychopathology, risk

## Abstract

Although high body mass index (BMI) alone does not invariably lead to body dissatisfaction (BD) and BD alone does not invariably lead to eating pathology (EP), research has suggested that there are clear relationships between each predictor and its respective criterion. We have a limited understanding of the factors that explain why some women at higher risk for BD (because of their BMI) do not report being dissatisfied with their bodies and why some women who are highly dissatisfied, do not engage in pathological eating behaviors. The present study examined such factors. A university sample of New Zealand women (*N* = 166) completed the Personality Assessment Inventory (Morey, 1991) and questionnaires measuring BD and EP. The tendency to report lower BD than would be predicted by one’s BMI, and the tendency to report lower EP than would be expected based on one’s BD, were characterized by lower overall distress (i.e., lower levels of anxiety and depression) and greater mood stability compared to those who followed the predicted outcome. Greater understanding of the factors that protect high-risk women from BD and EP may contribute to prevention and intervention strategies.

## Introduction

There are well-established links between body mass index (BMI) and body dissatisfaction (BD), and between BD and eating pathology (EP). Although BD is often described as a normative experience (Rodin et al., 1985), BMI has consistently been found to be a strong risk factor in increased BD (e.g., Stice and Whitenton, 2002). Extreme BD can be detrimental to one’s emotional wellbeing and is related to a variety of negative outcomes, such as low self-esteem, depressive symptoms, binge eating, reduced physical activity and extreme weight control behaviors (Johnson and Wardle, 2005; Neumark-Sztainer et al., 2006; van den Berg and Neumark-Sztainer, 2007). However, not all women with a high BMI are dissatisfied with their bodies. There are lingering gaps in the literature that do not address why some women with relatively high BMIs may have low BD, and an understanding of such potential protective factors is greatly needed.

In turn, BD is a consistent and robust predictor of EP (e.g., Stice and Shaw, 2002). Although BD is widespread, eating disorders (EDs) are relatively rare and not all women who are dissatisfied have or will develop an ED (Polivy and Herman, 2002). Research into the characteristics of women highly dissatisfied with their bodies, but who are not engaging in pathological eating behaviors is rare but important. Moderator research has shed some light on the factors that accentuate the relationship between BD and EP. For example among women, perfectionism, body surveillance, low self-compassion, neuroticism, low well-being; anxiety, depression, low self-esteem, and having a family member or friend with an ED, have each been found to moderate the relationship between BD and EP (Twamley and Davis, 1999; Tylka, 2004; Downey and Chang, 2007; Brannan and Petrie, 2011; Juarascio et al., 2011; Stutts and Blomquist, 2018). However, to our knowledge, no research has specifically looked at the characteristics of those with low levels of EP relative to their BD.

Certain personality factors might help identify individuals at high risk for developing an ED. In their review, Vitousek and Manke (1994) reported that neurotic personality traits such as obsessionality, dependency, compliance and anxiety tend to precede BD and EDs. Similarly, Anderluh et al. (2003) found retrospective reports of obsessive-compulsive personality traits during childhood were significantly higher for women with EDs than healthy controls. Research has demonstrated that high neuroticism is related to increased BD beyond the influence of BMI (MacNeill et al., 2017), and Dalley et al. (2009) reported an interaction between BMI and neuroticism, whereby increased BMI and neuroticism led to higher levels of BD. Although personality is likely to play a causal role in the development of EP, it is also likely that certain traits modify the course of ED development (Podar et al., 1999).

Studies that have examined the personality traits associated with EP have most commonly measured personality with the Eysenck Personality Questionnaire (Wade et al., 1995), Multidimensional Personality Questionnaire (Leon et al., 1995; Pryor and Wiederman, 1996) and the Minnesota Multiphasic Personality Inventory (Cachelin et al., 1997; Exterkate et al., 2007). Some researchers have used the Personality Assessment Inventory (PAI; Morey, 1991) among ED populations (e.g., Tasca et al., 2002; Bean et al., 2005; Tasca et al., 2009; Lampard et al., 2013; MacGregor and Lamborn, 2014); however, to our knowledge, no researchers to date have used the PAI to examine potential protective factors for EP. The PAI is considered a substantial improvement over other personality tests, has strong psychometric properties and, as such, has grown in clinical popularity (Helmes, 1993; Blais et al., 2010). In particular, the PAI addressed the importance of discriminant and convergent validity in personality measures and focused on both empirical and theoretical knowledge on clinical constructs (Morey, 2003). Furthermore, the PAI measures different aspects of each clinical construct in the subscale as well as their severity levels (Morey, 2003). For example, the Anxiety scale is made up three subscales: Cognitive, Affective and Physiological, which enables comprehensive and broad assessment of psychopathology and its intensity. Moreover, the PAI is relatively brief, captures much information and only requires a fourth-grade reading level, making this both less demanding and more accessible for respondents, researchers and clinicians.

The aim of the present study was to examine the personality and psychopathology characteristics of women who deviated from the expected relationships between BMI and BD and between BD and EP, through using a well validated and comprehensive personality measure. We reasoned that, if BMI is a predictor of BD, and BD is a predictor of EP, it would be useful to study those women who deviated from these predicted relationships, and what personality and psychopathology traits might predict such deviation. By using a broadband personality measure, we will have a useful starting point to elucidate the characteristics of these women and the personality and clinical factors that might lessen risk. Understanding the characteristics of women who deviate from these typical relationships may help clarify what makes some women less vulnerable to BD and EP. As this study was exploratory, no *a priori* hypotheses were made.

## Materials and Methods

### Participants

In total, 186 women aged between 18 and 40 and recruited from a university in New Zealand participated in this study. This study focused on young adult women because of the relatively higher prevalence of BD and EP in this demographic category (Berg et al., 2009) and because the PAI is suitable for those 18 years and above. Based on validity cut-offs of the PAI, data from 20 participants were excluded from the study and were not included in any of the analyses. This proportion of exclusions based on profile validity was similar to that found in the PAI development study with a non-clinical sample (Morey, 1991). A further six participants had incomplete datasets which meant that, using listwise deletion, the smallest sample size for any analysis was 160.

The mean BMI of the sample was 22.83 (*SD* = 3.45); 70% were in the normal weight range, and 6.7, 18.7, and 3.6% fell in the underweight, overweight, and obese ranges, respectively (National Heart Lung and Blood Institute et al., 1998). These BMI percentages were different from the population statistics of New Zealand from 2011 to 2013 where 2% were underweight, 44% were healthy weight, 26% were overweight, and 28% were obese (Ministry of Health, 2015)^1^. In terms of ethnicity, 78.9% identified as New Zealand European, 13.3% Asian/part Asian, 3.0% New Zealand Mâori, 1.2% Pacific Island/Part Pacific Island, and 3.6% other. This is slightly different to the census data of Christchurch, New Zealand where 83.9% identified as NZ European, 9.4% as Asian/part Asian, 8.5% as New Zealand Mâori, 3.1% as Pacific Island/Part Pacific Island and 4.8% as Other (Stats NZ, 2013).

### Measures

#### Demographic Information

A questionnaire assessed age, ethnicity, occupation, mother and father/caregiver occupation, self-reported height and weight.

#### Body Dissatisfaction

The Body Shape Questionnaire (BSQ; Cooper et al., 1987) is a 34-item measure of how individuals have felt about their appearance over the past 4 weeks. It is a commonly used and well-established measure of satisfaction and concern with body shape using a 6-point response format ranging from “never” to “always” (sample item: “Have you felt excessively large and rounded?”). Higher total scores are indicative of greater body shape dissatisfaction. The Cronbach’s alpha in this sample was 0.95.

The group-administered version of the Body Image Assessment (BIA; Williams et al., 2001) is a figural rating scale containing nine silhouettes ranging from very thin to very large presented in random order. Participants select the figure that best represents their current body size and the figure that best represents their ideal body size, in order to derive a discrepancy score. The group-administered version differs from the original in that the silhouettes are randomly presented on a piece of paper as opposed to individual cards. Test–retest reliability for the individually administered version was 0.90 for CBS and 0.71 for IBS (Williamson et al., 1989). Williams et al. (2001) reported similar psychometric properties for the group-administered version.

The Body Esteem Scale (BES; Franzoi and Shields, 1984) is a 35-item self-report measure of how an individual feels about parts of his or her body on a 5-point scale (from “Have strong negative feelings” to “Have strong positive feelings”). Lower scores on this scale are indicative of poorer body esteem. In this study, we only used the 10-item weight concern subscale (body parts for this subscale included: waist, thighs and figure). Researchers have reported adequate score reliability (alpha = 0.78–0.87) and test–retest reliability (*r* = 0.75–0.81 for women) (Franzoi and Shields, 1984; Franzoi, 1994). Cronbach’s alpha for scores on this subscale, for this sample, was 0.90.

#### Eating Pathology

The Eating Attitudes Test-26 (EAT-26; Garner et al., 1982), is a commonly used as a screening tool for dysfunctional eating attitudes and behaviors in non-clinical samples. The measure uses a 6-point scale ranging from “always” to “never” with items including “I feel terrified about being overweight” and “I give too much time and thought to food.” The EAT-26 is a shortened version based on the factor analysis of the EAT-40 and correlates highly with the original measure (*r* = 0.98) (Garner et al., 1982). Higher scores on the EAT-26 indicate greater EP. In the current study, Cronbach’s alpha was 0.89.

#### Personality

The Personality Assessment Inventory (PAI; Morey, 1991) is a 344-item self-report measure of adult personality and other clinical variables. The PAI contains eleven clinical scales: somatic complaints, anxiety, anxiety-related disorders, depression, mania, paranoia, schizophrenia, borderline features, antisocial features, alcohol problems, and drug problems. It also contains four validity scales: inconsistency, infrequency, negative impression and positive impression. The PAI has two interpersonal scales: dominance, which assesses control and independence in interpersonal relationships, and warmth, which measures supportiveness and empathy in relationships. The five treatment scales of the PAI were not examined in the current study. Morey (1991) reported good internal consistency for the PAI scales when using non-clinical, clinical and student samples, with median alphas of 0.81, 0.86, and 0.82 respectively. In the current study, Cronbach’s alphas ranged from 0.70–0.92 for the scales.

### Procedure

Participants were recruited through flyers and emails sent to various university departments and through an undergraduate Psychology department participant pool. Each participant gave informed consent to participate in this study, and questionnaires were group administered at scheduled times. Each participant completed the same paper questionnaire booklet, with questionnaires in the same order, in a spacious classroom. Participants had a space between them and the person next to them in order to ensure discretion. Height and weight were self-reported, but scales and a stadiometer were placed in a discrete part of the room for those participants who did not know their height and weight. Confidentiality was assured and participants were debriefed at the end of the study. This study was approved by the University of Canterbury Human Ethics Committee before testing commenced.

### Statistical Analyses

Data analyses were performed using SPSS version 25. Descriptive statistics were calculated to determine the sample composition. We performed regression analyses (predicting BD from BMI, and EP from BD) and then created residual scores for each participant (i.e., the degree to which they deviated from the predicted relationships). A positive residual score for the BMI to BD relationship would mean that an individual reported greater BD than would be predicted, based on her BMI; a negative residual would mean the opposite, and the same interpretative logic would apply to the relationship between BD and EP. Then we studied how those residual scores were related to the personality and psychopathology variables measured by the PAI. Independent sample *t*-tests and analysis of covariances (ANCOVAs; controlling for Positive and Negative Impression Management) were used to test for group differences between the high residual scorers and low residual scorers for both the BMI to BD and BD to EP relationships. Positive (PIM) and negative impression management (NIM) are two forms of response distortion. Although in a non-clinical setting there are no obvious motives for positive or negative distortion (in contrast with clinical or forensic settings), controlling for NIM and PIM allows one to see how the relationships change. In some situations, controlling for NIM and PIM may improve the validity of the other scores (Edens and Ruiz, 2008). For the *t*-tests, we tested the quality of variance assumption and if a specific variable did not meet this assumption, we reported the test that did not assume normality. For the ANCOVAS, the homogeneity of regression assumption was violated for Antisocial Features, Drug Problems and Warmth in the BMI-BD analysis, and for Dominance in the BD-EP analysis, therefore we did not perform an ANCOVA for those variables.

## Results

### Composite BD

Following the recommendations of Thompson (2004), we used multiple measures of BD to ensure that various dimensions of BD were being examined. However, because the three measures used (the BSQ, the BES, and the discrepancy score from the BIA) were highly correlated, we first conducted a principal components analysis to examine if these measures produced one component and, if so, to combine these into a composite variable for subsequent analyses. The Kaiser–Meyer–Olkin value for this PCA was 0.72, suggesting that data were moderately suitable for factor analysis (Kaiser, 1974). As expected, there was one eigenvalue greater than 1.00 (2.39) and the scree plot also indicated a one-component solution. We calculated this composite variable using the regression method and used it in the remainder of the analyses.

### Predictions of BD and EP

In the first regression analysis, BMI significantly predicted BD scores *F*(1,162) = 56.81, *R^2^* = 0.26; *p* < 0.01. In the second regression analysis, BD significantly predicted EP scores *F*(1,163) = 56.62, *R^2^* = 0.27; *p* < 0.01. Subsequent analyses examined the relationship between standardized residual scores and personality variables.

### Interrelationships Between Regression Residuals and Personality Variables

Table 1 displays the zero-order [Pearson] correlations between the personality and clinical variables of the PAI, and the residual scores from the two regression analyses. The BMI-BD residuals had significant and positive correlations with Somatic Complaints, Anxiety, Anxiety-Related Disorders, Depression, Paranoia, Schizophrenia, Borderline Features, Alcohol Problems, and NIM. These residuals also had significant but negative correlations with Dominance, Warmth, and PIM. We then calculated partial correlations, controlling for both NIM and PIM. In those analyses, five predictor variables were statistically significant: Anxiety, *r*(156) = 0.20, *p* = 0.012; Mania, *r*(156) = -0.20, *p* = 0.014; Borderline Features, *r*(156) = 0.19, *p* = 0.018; Antisocial Features, *r*(156) = -0.20, *p* = 0.011; and Dominance, *r*(156) = -0.27, *p* = 0.001.

**Table 1:** Zero-order correlations between personality variables and BMI-BD and BD-EP residuals, and Cronbach’s alpha for each variable (*N* = 160).

	α	BMI-BD residuals	BD- EP residuals
Somatic Complaints	0.88	0.21**	0.21**
Anxiety	0.92	0.36**	0.36**
Anxiety Related Disorders	0.86	0.34**	0.38**
Depression	0.91	0.37**	0.24**
Mania	0.84	0.01	0.26**
Paranoia	0.88	0.28**	0.15
Schizophrenia	0.88	0.20*	0.18*
Borderline Features	0.90	0.38**	0.24**
Antisocial Features	0.84	-0.03	-0.03
Alcohol Problems	0.86	0.16*	-0.08
Drug Problems	0.80	0.04	-0.01
Dominance	0.80	-0.31**	-0.10
Warmth	0.80	-0.26**	-0.08
PIM	0.75	-0.31**	-0.26**
NIM	0.70	0.28**	0.15


*^∗^p < 0.05. ^∗∗^p < 0.01; PIM, positive impression management; NIM, negative impression management.*

For the BD-EP residuals, the zero-order correlations were similar to the BMI-BD residuals. There were significant positive correlations with Somatic Complaints, Anxiety, Anxiety-Related Disorders, Depression, Mania, Schizophrenia and Borderline Features, and a significant negative relationship with PIM. In the partial correlation analyses, four individual variables correlated significantly with the residual scores, after controlling for NIM and PIM. These were Anxiety, *r*(157) = 0.25, *p* = 0.001, Anxiety-Related Disorders, *r*(157) = 0.29, *p* < 0.001; Mania, *r*(157) = 0.17, *p* = 0.037; and Alcohol Problems, *r*(157) = -0.18, *p* = 0.031.

### Group Differences for BMI-BD Residuals

The two groups created based on their distance from the residual mean, described earlier, were used to examine the personality and clinical differences of individuals with more extreme residuals from the BMI-BD regression model. The first subgroup comprised those individuals that scored one SD or more above the residual mean; in other words, they scored substantially higher on BD relative to their BMI. This group was referred to as the *high residual group* (*n* = 27). The second group comprised those that scored one SD or more below the residual mean, meaning that they scored low on BD relative to their BMI. This group was called the *low residual group (n* = 31). As expected, the high residual group had significantly greater scores on BD, *t*(56) = -21.32, *p* < 0.001, as well as on EP, *t*(56) = -5.88, *p* < 0.001. There was no significant difference in BMI, *t*(56) = 0.43, *p* = 0.668. We then compared the personality and clinical characteristics of the low residual group and high residual group using independent samples *t-*tests. The high residual group had significantly greater scores on Somatic Complaints, Anxiety, Anxiety-Related Disorders, Depression, Paranoia, Borderline Features and NIM; and lower scores on Dominance, Warmth and PIM, than the low residual group. The results of these group comparisons are presented in Table 2. Cohen’s *d* effect sizes ranged from medium to large.

**Table 2:** Comparisons of high and low residual groups for BMI-BD analysis.

Variable	High	Low	*t*-value	Effect size (*d*)
	(*n* = 27)	(*n* = 31)		
Somatic Complaints	53.22 (10.73)	48.84 (8.71)	-1.72*	-0.45
Anxiety	61.30 (11.71)	49.94 (8.36)	-4.20**	-1.12
Anxiety Related Disorders	58.04 (11.75)	47.52 (9.22)	-3.82**	-1.00
Depression	58.48 (13.30)	48.03 (11.01)	-3.27**	-0.86
Mania	51.89 (9.17)	51.94 (10.31)	0.02	0.01
Paranoia	53.89 (8.03)	47.94 (10.13)	-2.45**	-0.65
Schizophrenia	49.81 (11.02)	53.15 (12.46)	1.08	0.28
Borderline Features	60.48 (9.61)	50.23 (10.37)	-3.91**	-1.03
Antisocial Features	51.59 (6.99)	54.19 (11.30)	1.04	0.28
Alcohol Problems	54.41 (10.27)	51.61 (10.92)	-0.10	-0.26
Drug Problems	50.89 (8.79)	48.87 (8.70)	-0.44	-0.23
Dominance	41.81 (11.43)	52.29 (9.09)	3.89**	1.01
Warmth	48.00 (9.75)	54.32 (9.26)	2.53**	0.66
PIM	41.85 (10.46)	51.35 (9.97)	3.54**	0.93
NIM	54.00 (10.10)	48.81 (7.05)	-2.24*	-0.60


*Low, residuals 1 SD below regression line; High, residuals 1 SD above regression line; ^∗^*p* < 0.05. ^∗∗^*p* < 0.01; PIM, positive impression management; NIM, negative impression management.*

As a similar follow-up analysis, we performed the same mean comparisons while also controlling for both NIM and PIM scores (using ANCOVA). After controlling for those two scales, there were only statistically significant main effects for Anxiety, *F*(1,54) = 4.90, *p* = 0.031, $\eta_{p}^{2}$ = 0.083; Antisocial Features, *F*(1,54) = 8.90, *p* = 0.004, $\eta_{p}^{2}$ = 0.142, and Dominance, *F*(1,54) = 10.72, *p* = 0.002, $\eta_{p}^{2}$ = 0.166. The adjusted means showed the same pattern as the unadjusted means. That is, the high residual group was higher on Anxiety, and lower on both Antisocial Features and Dominance.

### Group Differences for BD-EP Residuals

We also used residuals for the BD-EP regression analysis to group participants into two subgroups: the low residual group (*n* = 21) and the high residual group (*n* = 23). As expected, the high residual group had significantly greater scores on EP, *t*(42) = -11.41, *p* < 0.001. There were no significant differences between the high and low group for BD or BMI, *t*(42) = 1.35, *p* = 0.198, and *t*(41) = 1.39, *p* = 0.172, respectively. The high residual group scored significantly higher on Somatic Complaints, Anxiety, Anxiety-Related Disorders, Mania and Borderline Features; and lower on PIM, than the low residual group. Table 3 reports the comparisons between these two groups. Cohen’s *d*s ranged from medium to large. As above, we also performed ANCOVAs, controlling for both NIM and PIM. In those analyses, the only variable on which there was a statistically significant group difference (after controlling for NIM and PIM) was Mania, *F*(1,40) = 6.71, *p* = 0.013, $\eta_{p}^{2}$ = 0.144. The direction of the difference for adjusted means was the same as for unadjusted means (the high residual group scored higher).

**Table 3:** Comparisons of high and low residual groups for BD-EP analysis.

Variable	High	Low	*t* value	Effect size (*d*)
	(*n* = 23)	(*n* = 21)		
Somatic Complaints	57.35 (9.56)	51.67 (8.62)	-2.02*	-0.62
Anxiety	65.61 (11.01)	54.29 (11.57)	-3.33**	-1.00
Anxiety Related Disorders	64.48 (13.95)	51.71 (13.69)	-3.06**	-0.92
Depression	60.04 (13.09)	53.62 (10.00)	-1.84	-0.55
Mania	58.17 (10.57)	48.38 (7.07)	-3.64**	-0.55
Paranoia	56.17 (10.90)	51.86 (8.07)	-1.48	-0.45
Schizophrenia	56.04 (13.31)	51.43 (12.19)	-1.20	-0.36
Borderline Features	64.70 (9.29)	56.86 (9.29)	-2.80*	-0.84
Antisocial Features	54.48 (8.38)	53.00 (6.73)	-0.64	-0.19
Alcohol Problems	51.26 (8.14)	52.95 (7.27)	0.72	0.22
Drug Problems	48.96 (7.58)	48.95 (6.77)	<0.01	<0.01
Dominance	46.30 (11.48)	45.57 (12.34)	-0.20	-0.06
Warmth	49.74 (8.69)	50.10 (10.53)	0.12	0.04
PIM	39.26 (8.15)	47.48 (11.11)	2.82**	0.84
NIM	56.35 (11.71)	52.05 (7.94)	-1.44	-0.43


*Low, residuals 1 SD below regression line; High, residuals 1 SD above regression line; ^∗^p < 0.05. ^∗∗^p < 0.01; PIM, positive impression management; NIM, negative impression management.*

## Discussion

In this study, we used the PAI scales to determine the characteristics of women who deviated from the expected relationship between BMI and BD and the relationship between BD and EP. To our knowledge, this study is the first to examine the PAI in relation to potential risk factors for EP and BD. Women in this sample exhibited similar personality features, as measured by the PAI, to the non-clinical standardization sample (Morey, 1991), demonstrating the comparability of this sample to the original sample.

The two data analytic strategies were similar and complementary, but not completely redundant. Treating the dependent variables as continuous allowed us to use the whole sample, which potentially made statistical tests more powerful. However, limiting the analyses to the more extreme groups (plus or minus one SD) allowed us to focus on more extreme deviation from predicted relationships.

### Characteristics of Women With Lower or Higher BD Than Predicted by BMI

The two methods produced similar results when examining the relationship with PAI scales at the univariate level. That is, for both the zero-order correlations and the *t*-tests, several variables (Somatic Complaints, Anxiety, Anxiety-Related Disorders, Depression, Paranoia, Borderline Features, Dominance, and Warmth by both methods) appeared to be related to the tendency to score higher or lower on BD than predicted by BMI. When controlling for NIM and PIM, using partial correlation, five scales still predicted the dependent variable. Two of the same scales, Anxiety and Dominance, were statistically significant in the ANCOVAs. Interestingly, the Antisocial Features scale only became statistically significant after controlling for NIM and PIM.

The fact that several variables turned up in the univariate analyses, when not controlling for anything else, may be due to conceptual overlap between the scales sharing the common theme of subjective distress (Morey, 1991). Here we will focus primarily on interpreting the smaller subset of scales that were statistically significant predictors in both analyses when controlling for NIM and PIM.

Women scoring high on the Anxiety scales are characterized by heightened sense of insecurity and self-doubt (Morey, 1991). These women may also be more likely to be obsessional and perfectionistic, possibly accompanied by high, unrealistic standards of thinness. Women who scored lower on this scale and were in the low residual group may have been more likely to be calm and optimistic, and possibly less hypervigilant about their weight. As such, they may have been less inclined to worry about the consequences of a high BMI or not meeting the socially prescribed ideal of thinness.

On an interpersonal level, the finding that high dominance was also characteristic of the low residual group (and that there was a negative correlation with the residual variable) may reflect a greater self-assurance, assertiveness, and confidence in their identity, among the women who reported less BD than would be predicted by their BMI. Consistent with this confidence, these women may be less likely to be anxious about or dissatisfied with their weight.

An interesting finding was the statistically significant *negative* correlation with Mania, after controlling for NIM and PIM. This finding was particularly interesting given that the correlation was in the opposite direction in the subsequent analysis (for the BD-EP relationship). In the ANCOVAs, the direction of the effect was the same (the low residual group had a higher score), but the difference was not statistically significant, *F*(1,54) = 2.471, *p* = 0.122. Thus, the finding did not appear across both analyses but, in this [non-clinical] sample, higher scores on Mania may be indicative of increased experiences of positive emotionality, alongside a lack of sustained attention to one’s body, which may protect against BD.

Considering these results together, it appears that low levels of general anxiety, perhaps reflected in their secure interpersonal relationships, may best characterize the group of women with lower BD than predicted by their BMI. These individuals may have healthy self-esteem, emotionally stability, and healthy relationships, which may be associated with feeling comfortable with body weight and less likely to feel pressure to ascribe to social standards of thinness.

### Characteristics of Women With Lower or Higher EP Than Predicted by BD

As with the previous analyses, several variables (Anxiety, Anxiety-Related Disorders, Mania, Borderline Features, and Somatic Complaints) were significantly associated with the residual variable, when considered individually. This pattern may again be indicative of a general distress factor. However, a much smaller number of variables (Anxiety, Anxiety-Related Disorders, Mania, and Alcohol Problems) had statistically significant partial correlations when controlling for NIM and PIM, and the high and low residual group only differed significantly on one variable (Mania). It is noteworthy that these comparisons (the ANCOVAs) had less statistical power than the previous set of analyses (for women with lower or higher BD than predicted by BMI) because the numbers of participants in each group were smaller (although they likely had more statistical power than the *t*-tests). However, the only other scale that approached statistical significance was Anxiety, *F*(1,40) = 3.554, *p* = 0.067, $\eta_{p}^{2}$ = 0.082. The direction of the effect was also the same as when not controlling for NIM and PIM (the high residual group scored higher).

The finding that Mania scores were lower among the low residual group (and the positive correlation) may reflect the mood stability, affect regulation skills and secure sense of self among individuals with less EP than predicted by their BD (Morey, 1991). Mood stability may be associated with less EP because, although dissatisfied, a mood-stable woman may have the ability to cope adaptively with her dissatisfaction without engaging in pathological eating behaviors.

The relationship with the Anxiety scale was statistically significant in the zero-order and partial correlations, and in the *t*-test, but only marginally significant in the ANCOVAs (although caution should be applied when discussing marginal significance; see Pritschet et al., 2016). A relationship between anxiety and EP is well established (Keel et al., 2005), and the present results suggest that there may also be a relationship with the tendency to have more or less EP than predicted by BD. Individuals with a lower EP may have low anxiety characterized by a strong and optimistic sense of self and by employing adaptable ways to cope with stress (Morey, 1991). Such calmness and security may be associated with protection from developing and engaging in pathological eating behaviors because, although dissatisfied with their bodies, these women may not view losing weight as a means of increasing their self-confidence as may be the case for women high in anxiety. This finding is perhaps consistent with the findings of Twamley and Davis (1999) that self-esteem, a construct related to Morey’s (1991) description of anxiety on this scale, may buffer the effects of BD on EP among women.

As with the previous analyses, there was one variable, Alcohol Problems, that was a significant correlate (*r* = -0.171) of the residual score in the partial correlations. The fact that the correlation was negative may initially seem surprising and counter-intuitive. However, it is possible that women with higher residual scores (have more EP than predicted by their BD) avoid drinking because of the calories in alcohol. Indeed, Stock et al. (2002) found that individuals with restrictive eating problems report less substance use than the general (non-clinical) population. Looking at these findings collectively, it seems that the women who had less EP than predicted by their level of BD also exhibited less emotional distress (e.g., mood lability and anxiety) and may have been relatively emotionally healthy in general.

### Limitations

A significant limitation of this study was the cross-sectional design. It is important to consider the need for longitudinal research to corroborate these findings. It is also possible that a third uncontrolled variable was responsible for some significant findings, or that causal relationships may exist in the opposite direction (e.g., EP may cause anxiety or mood instability). We also only examined personality and clinical factors, as measured by the PAI, and there may have been other social or physical factors that play similar roles. For example, variation in BMI can be related to both adiposity and lean body mass. Some women with a larger BMI may have had more lean body mass and less body fat; thus, they experience less BD. Moreover, the women studied here may not be representative of the New Zealand population given the difference between the sample ethnicity and BMI percentages, and New Zealand population statistics. A further limitation is the relatively small sample size which left some of the statistical tests with reduced statistical power. Finally, BMI data were based on participant self-report. Although scales and a stadiometer were available for those who did not know their measurements, and self-reported and actual BMI tend to be highly correlated (Spencer et al., 2001), it would have been preferable to get anthropometric measurement for all participants.

Despite these limitations, this research highlighted a variety of personality and clinical variables that were related to a tendency to experience more or less BD than predicted by one’s BMI, and the same regarding experiencing more or less EP than predicted by one’s BD. Some of these factors may reflect features that could protect women from developing either BD or EP. The current study has important implications in terms of understanding the personality and clinical features of the women in these high-risk groups. Furthermore, this research points to potential targets in interrupting the development of EP, whether that be at the high BMI or the high BD stage.

Based on the collective analyses, women at who reported less BD or EP than predicted were typically less anxious and less emotionally labile than were the women who reported relatively more BD or EP than predicted (as measured by, for example, Anxiety, Anxiety-Related Disorders, Manic features). This trend suggests that targeting these factors among women with high BMIs and women with high BD may influence the related negative outcome. Longitudinal research is needed to corroborate the role of these factors among women who are in these high-risk groups. Such research would enable a greater understanding of why some women develop BD and EP and why some women do not and could be utilized in intervention planning. Moreover, research should address additional factors and explore how they may characterize these high-risk groups. If longitudinal research supports these findings, interventions could target these factors with women who are in these high-risk groups.

## Ethics Statement

This study was carried out in accordance with the recommendations of University of Canterbury Human Ethics Committee with written informed consent from all subjects. All subjects gave written informed consent in accordance with the Declaration of Helsinki. The protocol was approved by the University of Canterbury Human Ethics Committee.

## Author Contributions

JR contributed to the conceptualization of the study, performed the statistical analyses, interpretation of the data, and drafted the manuscript. DG contributed to the conceptualization of the study, performed the statistical analyses, interpretation of the data, and drafting of the manuscript. JL contributed to the conceptualization of the study and drafting of the manuscript. All authors read and approved the final manuscript.

## Conflict of Interest Statement

The authors declare that the research was conducted in the absence of any commercial or financial relationships that could be construed as a potential conflict of interest.

## Footnotes

1 These population statistics were unweighted averages taken from the BMI percentages of age groups corresponding to the participants in the current study.
